# Targeting Cancer Cell Ferroptosis to Reverse Immune Checkpoint Inhibitor Therapy Resistance

**DOI:** 10.3389/fcell.2022.818453

**Published:** 2022-03-24

**Authors:** Jingjing Deng, Mei Zhou, Tingting Liao, Wenlong Kuang, Hui Xia, Zhengrong Yin, Qi Tan, Yumei Li, Siwei Song, E Zhou, Yang Jin

**Affiliations:** ^1^ Department of Respiratory and Critical Care Medicine, NHC Key Laboratory of Pulmonary Diseases, Hubei Clinical Research Center for Respiratory Diseases, Union Hospital, Tongji Medical College, Huazhong University of Science and Technology, Wuhan, China; ^2^ Department of Cardiovascular Medicine, Union Hospital, Tongji Medical College, Huazhong University of Science and Technology, Wuhan, China

**Keywords:** immune checkpoints therapy, ferroptosis, apoptosis, cell death, immunogenic cell death, IFN-interferon, Tyro 3

## Abstract

In recent years, cancer therapies using immune checkpoint inhibitors (ICIs) have achieved meaningful success, with patients with advanced tumors presenting longer survival times and better quality of life. However, several patients still do not exhibit good clinical outcomes for ICI therapy due to low sensitivity. To solve this, researchers have focused on identifying the cellular and molecular mechanisms underlying resistance to ICI therapy. ICI therapy induces apoptosis, which is the most frequent regulated cell death (RCD) but lacks immunogenicity and is regarded as an “immune silent” cell death. Ferroptosis, a unique type of non-apoptotic-RCD, has been preliminarily identified as an immunogenic cell death (ICD), stimulating tumor-antigen-specific immune responses and augmenting anti-tumor immune effects. However, ferroptosis has rarely been used in clinical practice. Present evidence strongly supports that the interferon-γ signaling pathway is at the crossroads of ICI therapy and ferroptosis. TYRO3, a receptor tyrosine kinase, is highly expressed in tumors and can induce anti-programmed cell death (PD)-ligand 1/PD-1 therapy resistance by limiting tumoral ferroptosis. Therefore, in this review, we summarize the clinical practice and effects of ICI therapy in various cancers. We also provide an overview of ferroptosis and report the molecular connections between cancer cell ferroptosis and ICI therapy, and discuss the possibility to reverse ICI therapy resistance by inducing cancer cell ferroptosis.

## 1 Introduction

Among the various immunotherapeutic strategies, immune checkpoint inhibitors (ICI) therapy has shown remarkable benefits in treating several cancer types. ICI therapy targets immunosuppressive molecules to enhance anti-tumor effects. Examples are the monoclonal antibodies (mAbs) for programmed cell death 1/ligand 1 (PD-1/PD-L1) and cytotoxic T-lymphocyte antigen 4 (CTLA-4), both of which suppress the cytotoxic effect of CD8 T cells on tumor cells. Based on ICI mechanism, it is regarded as a cure-all in the field of anti-cancer therapy. Indeed, ICI therapy has shown unprecedented efficacy. In a phase 3 clinical trial, ipilimumab plus nivolumab, each respectively targeting the CTLA-4 and PD-1 immune checkpoints for treating advanced melanoma, increased the overall survival (>5 years) to ∼52%, while 58 and 62% of patients experienced an objective response and durable response at 5 years, respectively (Larkin et al., 2019). However, some patients show low response to ICI therapy and, from basic research to clinical practice, researchers have tried to disclose the intrinsic mechanisms explaining this phenomenon. Havel et al. concluded that tumor genomics, host germline genetics, PD-L1 level, and the intrinsic features of the tumor microenvironment, as well as the gut microbiome, may be the main reasons for such low response to ICI therapy (Havel et al., 2019).

Ferroptosis, a concept first introduced in 2012, is an iron-dependent form of non-apoptotic cell death, morphologically characterized by necrotic morphology, dysmorphic small mitochondria with decreased crista, condensed membrane, and a ruptured outer membrane (Dixon et al., 2012). As a form of regulated cell death (RCD), ferroptosis can be activated or prevented according to different physical conditions in the cell environment. Ferroptosis has also been implicated in pathological cell death associated with degenerative diseases (e.g., Alzheimer’s, Huntington’s, and Parkinson’s diseases), carcinogenesis, stroke, intracerebral hemorrhage, traumatic brain injury, ischemia-reperfusion injury, and kidney degeneration in mammals (Stockwell et al., 2017). The role of ferroptosis in cancers is worth special attention. A number of cancer genes have been identified their various functions in regulating cancer cells ferroptosis. For example, certain studies indicate that p53 has both stimulative and inhibitory effects on cancer cells ferroptosis by regulating metabolism directly or through its transcriptional downstream molecules. Apoptosis is the mainstream method of cell death in most circumstances, and ICI therapy is no exception. However, due to the acquired or intrinsic resistance of cancer cells to apoptosis (Hassannia et al., 2019), the efficacy of most tumor therapies is restricted; thus, research has focused on other types of cell death, including ferroptosis. Additionally, the reliance of cancer cells on iron metabolism, reactive oxygen species (ROS) excess, or lack of glutathione may confer ferroptosis the potential to kill neoplastic cells.

Combination therapy with ferroptosis-inducing drugs may play a potential role in solving low sensitivity of ICIs monotherapy. Indeed, combining anti-PD1 mAbs with anti-CTLA-4 mAbs has been shown to increase ICI therapy sensitivity, and research into combining ICIs with other reagents is also a promising direction. As so, we hypothesize that combining ICIs with ferroptosis inducers could be a valid pathway for treating cancers. Regrettably, these ferroptosis inducers’ role in inducing ferroptosis has not been demonstrated clinically, thereby restricting the clinical application of ferroptosis.

However, the findings stated above urge us to review the molecular mechanisms linking cancer cell ferroptosis and ICI therapy, further discussing the possibility to combine ICI therapy with ferroptosis for overcoming low-sensitivity to ICIs from a theoretical perspective.

## 2 Clinical Application of ICI Therapy

Since the first ICI, ipilimumab, was approved for clinical use by the US Food and Drug Administration (FDA) in 2011, six other ICIs have been described. Thus, seven ICIs are currently in clinical use for cancer therapy, ipilimumab—targeting CTLA-4—pembrolizumab and nivolumab—targeting PD-1—and atezolizumab, avelumab, durvalumab, and cemiplimab, targeting PD-L1. The range of indications for these ICIs is quite extensive and their clinical efficacies for treating several common cancers (e.g., advanced melanoma, non-small-cell lung cancer, lymphoma, urothelial carcinoma, renal cell carcinoma, and bladder cancer) is shown in Table 1.

**TABLE 1:** The clinic data of ICIs for treating cancers.

	MOS (month)	PFS (month)	OS (%)	ORR (%)	CRR (%)
Melanoma					
Ipilimumab Larkin et al. (2019)	19.9	2.9	26	19	6
Nivolumab Larkin et al. (2019)	36.9	6.9	44	45	19
Ipilimumab plus nivolumab Larkin et al. (2019)	>60	11.5	52	58	22
NSCLC					
Nivolumab Rizvi et al. (2015) ^,*1^	12.2	2.3	51^#1^	19	1
Nivolumab plus ipilimumab Hellmann et al. (2019)	17.1	5.1	40^#2^	35.9	5.8
Pembrolizumab Mok et al. (2019) ^,*2^	16.7	5.4	39^#2^	27	NR
Atezolizumab Rittmeyer et al. (2017) ^,*2^	15.7	2.8	58^#1^	18	2
Cemiplimab Sezer et al. (2021) ^*3^	>48	8.2	50^#2^	39	2
Hodgkin’s lymphoma					
Nivolumab Ramchandren et al. (2019) ^,*4^	NR	NR	NR	84	67
Pembrolizumab Chen et al. (2019) ^,*4^	>24	13.7	100^#2^	71.9	27.6
Urothelial carcinoma					
Atezolizumab Galsky et al. (2020)	15.7	NR	NR	23	6
Renal cell carcinoma					
Nivolumab Motzer et al. (2015)	25	4.6	NR	25	1
Nivolumab plus ipilimumab Motzer et al. (2018)	>30	11.6	NR	42	9
Bladder cacer					
Atezolizumab Inman et al. (2017) ^,*2^	7.9	2.1	36^#1^	15	5

MOS, median overall survival; PFS, median progression-free survival; OS, overall survival rate; ORR, objective response rate; CRR, complete response rate; PRR, partial response rate; *1, data from Nonsquamous NSCLC; *2, data from patients with ≥1% PD-L1 expression on tumour cells or tumour-infiltrating immune cells; *3, data from patients with ≥50% PD-L1 expression on tumour cells; *4, data from phase 2 clinical trial; #1, overall survival at 12 months; #2, overall survival at 24 months; NR, not reported.

Beyond canonical immune checkpoints PD-1/PD-L1 and CTLA-4, the next generation immune checkpoints such as lymphocyte activation gene-3 (LAG-3), T cell immunoglobulin and mucin-domaincontaining-3 (TIM-3), T cell immunoglobulin and ITIM domain (TIGIT), B7 homolog 3 protein (B7-H3) and V-domain Ig suppressor of T cell activation (VISTA) demonstrate as promising therapeutic targets with the possibility to realize clinical application (Qin et al., 2019). A most recent clinical trial study shows that relatimab (a monoclonal antibody against LAG-3) plus nivolumab therapy achieved better clinical efficacy (10.1 months median progression-free survival) than nivolumab monotherapy (4.6 months) in treating advanced melanoma, along with higher adverse event rate naturally (Tawbi et al., 2022).

Collectively, cancer patients benefit more from ICIs than from traditional chemotherapy, as the median overall survival (MOS) and objective response rate (ORR) of ICI therapy nearly reach or exceed that of chemotherapy, with fewer adverse effects. However, the survival rate at 5 years and the median progression-free survival rate of patients registered so far have hindered the success of ICI therapy.

## 3 Too Mild to Kill Apoptosis: An Important Cause of ICI Insensitivity

Apoptosis is most frequently primed during cancer cell death in response to multiple drug treatment or spontaneous or stimulated immune cell attack. However, cancer cells often have an integrated system to evade apoptosis, which may block the function of ICIs.

### 3.1 T Cells Effectively Kill Cancer Cells via Apoptosis

In the process of anticancer immune response leading to the effective killing of cancer cells, a series of stepwise events (the cancer-immunity cycle) must be initiated (Chen and Mellman, 2013): 1) tumor antigens are released from tumor cells and recognized by antigen presenting cells (APCs) in local lymphatic tissues; 2) APCs present tumor antigens to specific cytotoxic T lymphocytes (CTLs) for recognition; 3) effector T cell responses against cancer-specific antigens are primed and activated; 4) the activated effector T cells traffic to and infiltrate tumor lesions; 5) the activated effector T cells recognize target cancer cells; 6) the activated effector T cells kill target cancer cells by the perforin-and-granzyme-mediated pathway or the Fas-mediated pathway (Berke, 1995).

**FIGURE 1:**
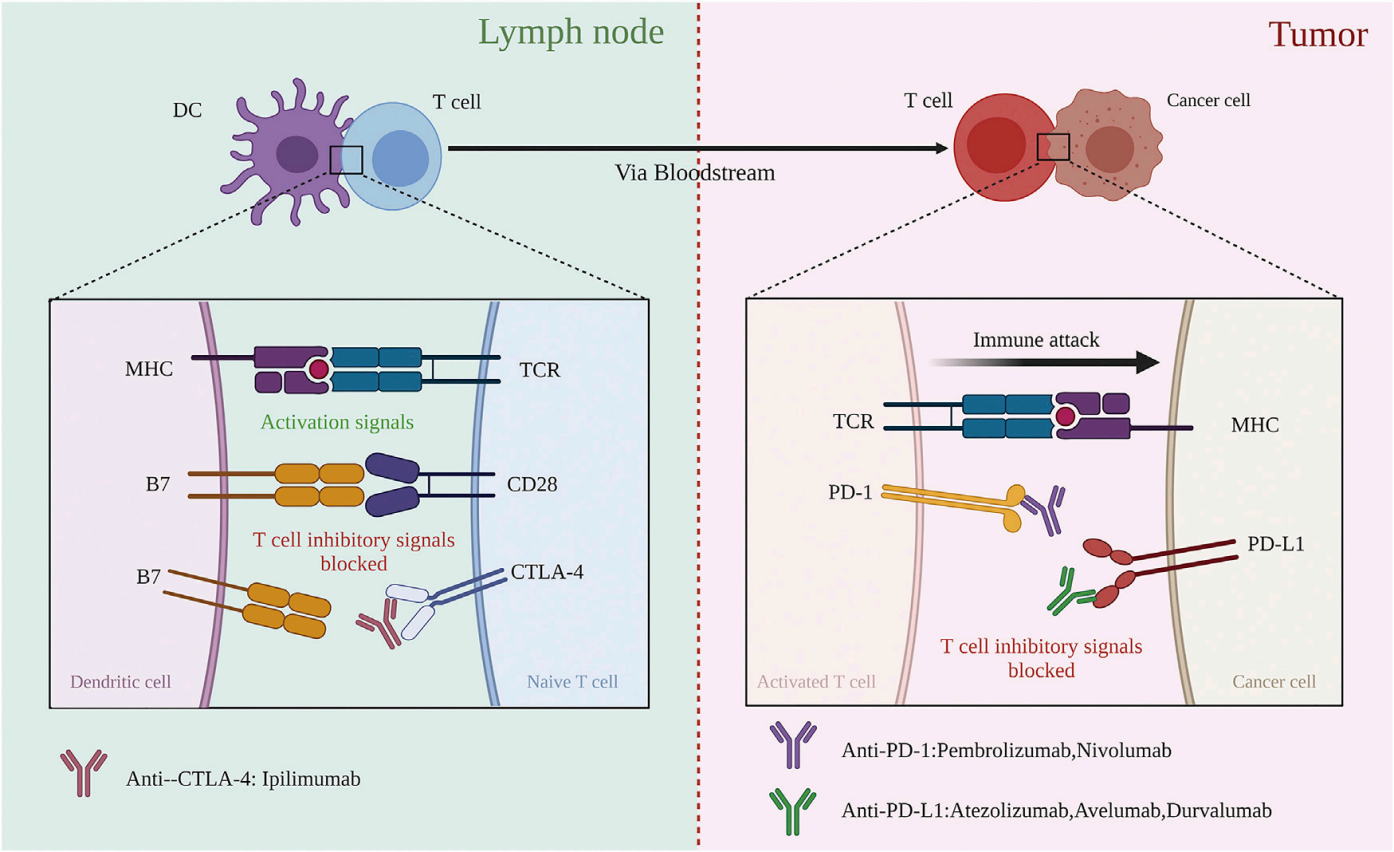
Blockade of CTLA-4 and PD-1 and PD-L1 to enhance antitumor response. (Left) Co-stimulation that CTLA-4 negatively regulates is required for initial activation of CD8 T cell during recognition of its specific tumour antigen, presented by an antigen-presented cell in the lymph node. Blocking CTLA-4 by anti-CTLA-4 antibodies could restore the intensity of co-stimulation. (Right) Activated CD8 T cells migrate to the tumor side based on recognizing specific tumor antigens expressed on the target cancer cell. Once recognized, the antitumor T cell response gets weak, with T cells expressing PD-1 and target cells expressing PD-L1. The negative regulation could be blocked by anti-PD-1 or anti-PD-L1 antibodies.

Many molecules participate in the process of antigen presentation, which includes several ligand-receptor interactions between T cells and APCs. The peptide major histocompatibility complex (MHC) I complexes expressed on APCs are recognized by T cell receptors (TCRs) along with CD4 molecules, consisting of the first signal. A series of ligands binds to multiple receptors, some of which delivering co-stimulatory signals and others inhibitory signals. In general, the same ligand(s) binds pairs of co-stimulatory/inhibitory receptors, such as CD28 and CTLA-4, therefore displaying distinct expression statuses; while the co-stimulatory receptor is usually expressed on naive or resting T cells, the inhibitory receptor is often upregulated after the activation of T cells. Family B7 is an important membrane-bound ligand family that binds co-stimulatory and inhibitory receptors (Pardoll, 2012). Notably, anti-CTLA-4 ICIs strengthen step (3), i.e., the activation of CTLs, by blocking the function of CTLA-4. Antigen-stimulated CTLs recognize the peptide-MHC I complexes expressed on cancer cells and then kill cancer cells. However, this process is frequently impaired due to the interaction of PD-1 and PD-L1, which induce CTLs death (Chen and Mellman, 2013). PD-1, an important immune checkpoint whose ligand, PD-L1, is frequently expressed on cancer cell membranes, mainly functions in step (6), killing target cancer cells (Figure 1).

Two pathways initiate apoptosis: the extrinsic and intrinsic pathways, mediated by cell death receptors on the cell surface and by mitochondria. As mentioned above, activated CD8 T cells mediate apoptosis through the perforin-and-granzyme-mediated pathway and Fas-mediated pathway, the latter being an extrinsic pathway. However, it remains undisclosed to which pathway category (intrinsic or extrinsic) the perforin-and-granzyme-mediated pathway belongs to.

Perforin, a 67 kDa multidomain protein coupled to the plasma membrane, can oligomerize to assemble pores that deliver the pro-apoptotic granzymes into the cytosol of the target cell (Law et al., 2010). Granzyme B is the most effective pro-apoptotic granzyme with the ability to cleave target cell proteins at the sites after aspartate residues by simulating caspases (Voskoboinik et al., 2015). Perforin-and-granzyme B-induced apoptosis can be regulated by mitochondria-dependent or mitochondria-independent pathways (MacDonald et al., 1999). Granzyme B typically activates one or more members of the BH3-only protein family in a mitochondria-dependent manner. When BH3 interacting-domain death agonists (BIDs) are activated above a crucial threshold, the inhibitory effect of the anti-apoptotic B-cell lymphoma-2 (BCL-2) family members can be interrupted, initiating the production of BCL-2-antagonist/killer-1 (BAK)– BCL-2-associated X protein (BAX) oligomers on mitochondrial outer membranes. Cytochrome c traverses from the mitochondria into the cytosol through these oligomers. The released cytochrome c binds to the adaptor molecule apoptotic peptidase activating factor 1 (APAF1) forming a complex, the apoptosome, which in turn activates caspase-9, the initiator caspase (Bock and Tait, 2020). It is worth mentioning that granzyme B is able to activate caspase-3 directly via a mitochondria-independent pathway (Kaiserman et al., 2006). In fact, human granzyme B activates the BID pathway to induce apoptosis, whereas mouse granzyme B induces apoptosis by directly activating caspase-3 (Kaiserman et al., 2006).

Death receptors are members of the tumor necrosis factor (TNF) receptor superfamily and comprise a subfamily characterized by an intracellular domain, the death domain (Igney and Krammer, 2002). When the Fas ligand (FasL) binds to Fas, the death domain attracts the intracellular adaptor protein Fas-associated death domain (FADD), which in turn recruits the inactive precursors of certain members of the caspase-protease family. The death-inducing signaling complex (DISC) assembled at the cytosolic face of several TNF receptor family members (Van Opdenbosch and Lamkanfi, 2019) then recruits caspase-8 to function as an initiator. Following this homo-activation of caspase-8, the caspase cascade reaction is initiated: the effector caspases, caspase-3/6/7, gain proteolytic activity after the active caspase-8 cuts the link between their large and small catalytic subunits (van Opdenbosch and Lamkanfi, 2019). The cleavage and activation of BID to generate truncated BID (tBID) mediated by caspase-8 connects the extrinsic apoptotic pathway to the intrinsic pathway (Bock and Tait, 2020). These pathways share the same effector caspases. The downstream molecules of effector caspases orchestrate the dismantling of diverse cell structures.

In summary, the series of proteolytic events described above generate the cell morphological changes characteristic of apoptosis, including membrane blebbing, cell shrinkage, the formation of ‘‘apoptotic bodies,’’ and chromosomal DNA fragmentation (Ramirez and Salvesen, 2018). For example, cleavage of the inhibitor of caspase-activated DNase (ICAD) releases caspase-activated DNase (CAD), which can then catalyze inter-nucleosomal DNA cleavage. Proteolysis of the effector Rho-associated coiled-coil containing protein kinase 1 (ROCK1) causes plasma membrane blebbing and nuclear fragmentation, while cleavage of tubulins and microtubule-associated motor proteins leads to changes in the microtubule cytoskeleton that may contribute to apoptotic body formation (Taylor et al., 2008). The cleavage of nuclear lamins mediated by caspases breaks the nuclear lamina, allowing nuclear fragmentation (Taylor et al., 2008).

### 3.2 Apoptosis is not Necessarily Immunogenic

The resistance of neoplastic cells to apoptosis is an important issue with relatively complete theoretical research. Frederik H. Igney and Peter H. Krammer reported complete tumor resistance to apoptosis in 2002. They considered the expression of anti-apoptotic proteins, inactivation of pro-apoptotic genes, alteration of the PI3K/AKT and p53 pathways, and altered survival signaling as the main resistant mechanisms (Igney and Krammer, 2002). In 2003, Wei Hu and John J. Kavanagh reviewed the anticancer therapy targeting the apoptotic pathway from a clinical perspective (Hu and Kavanagh, 2003). Later, Mohammad and co-workers discussed how Bcl-2 and myeloid cell leukemia-1 (Mcl-1) proteins, autophagy processes, necrosis and necroptosis, heat shock protein signaling, the proteasome pathway, epigenetic mechanisms, and aberrant nuclear export signaling could function as key resistant targets (Mohammad et al., 2015). Overall, these studies pointed out that tumor cells can overcome apoptosis in various ways. In fact, the development of cancer immunology over the past 2 decades has elucidated that apoptosis is not important in the activation of adaptive immunity, which is deemed as an “immune silent” process.

Dying cells are not silent objects; inversely, they have to interact with immune cells to be cleared. Dying cells release soluble chemo-attractants (designated “find-me signals”) as executioner caspases, which are activated during apoptosis. These signals commonly include adenosine triphosphate (ATP), uridine triphosphate (UTP), chemokine C-X3-C motif ligand 1 (CX3CL1), and sphingosine-1-phosphate (S1P) (Elliott and Ravichandran, 2016). The rapid, caspase-dependent exposure of phosphatidylserine (PtdSer) to the outer leaflet of the apoptotic cell plasma membrane is a canonical feature of apoptosis, which is part of the “eat-me signal” along with ligands recognized by phagocytes (Elliott and Ravichandran, 2016). Immunogenic cell death (ICD) is a form of cell death that induces an effective antigen-specific immune response through the activation of dendritic cells (DCs). Hallmarks include the exposure of calreticulin on the dying cell surface and the active release of ATP and high mobility group protein b1 (HMGB1) into the extracellular milieu (Wculek et al., 2020). After DCs engulf the released ATP and HMGB1, they acquire a mature phenotype. It is worth noting that ICD is not programmed cell death but a subtle successful dialogue between a dying cell and the immune system (Legrand et al., 2019). ICD theory demonstrates that the immunogenicity of cell death relies on a combination of antigenicity, provided by neoepitopes, and adjuvanticity, conferred by specific damage-associated molecular patterns (DAMPs) (Galluzzi et al., 2017). DAMPs include various molecules, including ATP, BCL-2, calreticulin, HMGB1, and heat-shock proteins (HSPs), most of them functioning as “find-me” or “eat-me” signals. Cancer cells often escape immunosurveillance because of their defects in the release of DAMPs.

In most cases, apoptosis cannot be considered as ICD because the apoptotic cells produce “eat-me” signals identified by phagocytes that mediate rapid phagocytosis, which is too rapid to activate a specific immune response (Nagata and Tanaka, 2017). Additionally, due to a caspase- and ROS-dependent mechanism leading to the oxidative inactivation of HMGB1, apoptosis is generally not considered immunogenic (Kroemer et al., 2013). However, under some chemotherapy and irradiation therapies apoptosis is immunogenic (Galluzzi et al., 2017). For example, when thapsigargin or tunicamycin were combined with cisplatin, the induced apoptosis was immunogenic (Martins et al., 2011). Moreover, chemotherapeutic drugs including mitoxantrone (MTX), oxaliplatin, cyclophosphamide, and bortezomib, and the γ-irradiation therapy, all of which inducing apoptosis, are ideal ICD inducers (Krysko et al., 2012).

The ability of selected cancer therapies (i.e., doxorubicin, MTX, and γ-irradiation) to induce ICD depends on their ability to induce endoplasmic reticulum (ER) stress and ROS production. Both ER stress and ROS production are essential for initiating intracellular danger-signaling pathways that govern ICD (Krysko et al., 2012). For instance, the immunogenicity of ICD was diminished in the presence of antioxidants (Panaretakis et al., 2009), indicating the importance of ROS for inducing ICD. However, it is not clear whether ICI-induced apoptosis is immunogenic, which is a question worth of further investigation.

### 3.3 Other Forms of ICD: Pyroptosis and Necroptosis

Necroptosis can be initiated in response to cellular stress, cellular damage, or infection through several means, including binding to extrinsic apoptotic death receptors, i.e., Toll-like receptors (TLRs) by TIR-domain-containing adapter-inducing interferon-β (TRIF), and in response to viral infections through Z-DNA-Binding Protein 1 (ZBP1) (Koren and Fuchs, 2021). Pyroptosis is primarily utilized by innate immune cells in response to cellular perturbations, damage, and pathogen-induced signals (Koren and Fuchs, 2021), triggered by the binding of pathogen-associated molecular patterns (PAMPs) and DAMPs, by pattern recognition receptors (PRRs) and inflammasomes (Rosenbaum et al., 2021). The mechanisms of pyroptosis and necroptosis have been thoroughly reviewed (e.g., Koren and Fuchs, 2021). Apoptosis, pyroptosis, and necroptosis highly crosstalk, while ferroptosis highly distinct from these three mechanisms.

Pyroptosis and necroptosis can activate pro-inflammatory signaling and promote antitumor immunity (Carneiro and El-Deiry, 2020). The inflammatory nature of pyroptosis is mediated by pore-forming gasdermin (GSDM) proteins that facilitate immune cell activation and infiltration by inducing the release of pro-inflammatory cytokines and immunogenic material following cell rupture (Loveless et al., 2021). In addition to the release of traditional immunogenic material, such as HMGB1 and ATP, pyroptosis includes the release of interleukin (IL)-1β and IL-18. Moreover, tumor cells undergoing pyroptosis generate large amounts of neoantigens that induce systemic immune response (Tan et al., 2021). Unlike pyroptosis, which is a primary cellular response following the sensing of potential damaging insults, necroptosis is mediated by mixed-lineage kinase domain like pseudokinases (MLKLs) and regarded as a backup cell death mechanism that is triggered when apoptosis is inhibited (Frank and Vince, 2019). Cancer cells can produce DAMPs (e.g., HMGB1, ATP, and IL-1α) and tumor antigens (TAs) during necroptosis. In a prophylactic tumor vaccination model, necroptotic cancer cells showed CD8^+^ T cell cross-priming, protection against challenge with live tumor cells, and a TA-specific IFN-γ response; in addition, DAMPs released from the necroptotic cancer cells activated DC maturation in co-culture, strongly indicating the immunogenic nature of necroptosis (Krysko et al., 2017).

## 4 Ferroptosis Potential in Cancer Therapy

### 4.1 Ferroptosis Overview

Ferroptosis was initially induced by the small molecule erastin (Dixon et al., 2012), which is an inhibitor of system xc^−^, consisting of SLC3A2 and SLC7A11 and taking up cystine by forming a glutamate/cysteine antiporter in the cell’s plasma membrane (Fearnhead et al., 2017). As the synthesis of glutathione (GSH) requires cysteine, erastin causes a decrease in GSH levels, making the cells vulnerable to oxidative damage. The other canonical way to trigger ferroptosis is the depletion of glutathione peroxidase 4 (GPX4), an enzyme able to detoxify hydroperoxides in complex lipids, even when they are inserted into membranes or lipoproteins (Brigelius-Flohé and Maiorino, 2013). Notably, GSH is the cofactor of GPX4. In the absence of GPX4, uncontrolled peroxidative attack on membrane lipids occurs through the accumulation of lipid radicals, lipid peroxy radicals, and alkoxyl radicals (Fearnhead et al., 2017). In ferroptosis, the production of these radicals is centered on the Fenton or Fenton-like reactions, in which the ferrous iron (Fe^2+^) is oxidized to the ferric iron (Fe^3+^) through reaction with hydrogen peroxide (H_2_O_2_), resulting in the formation of highly reactive hydroxyl radicals (OH^.^) (Shen et al., 2018; Hassannia et al., 2019; Tang et al., 2021). Although there are many lipids on the cell membrane, the free radicals that attack polyunsaturated fatty acids (PUFAs) on the plasma membrane seem to be the most important for ferroptosis (Kagan et al., 2017).

In addition to the non-enzymatic lipid peroxidation discussed above, there is an enzymatic lipid peroxidation mediated by the lipoxygenase (LOX) family. LOXs are non-heme iron-containing enzymes that directly catalyze esterified PUFAs to generate various lipid hydroperoxides directly (Zheng and Conrad, 2020). Because LOXs require iron during their catalytic process, they are regarded as iron-dependent enzymes. Iron can also regulate enzymatic lipid peroxidation.

**FIGURE 2:**
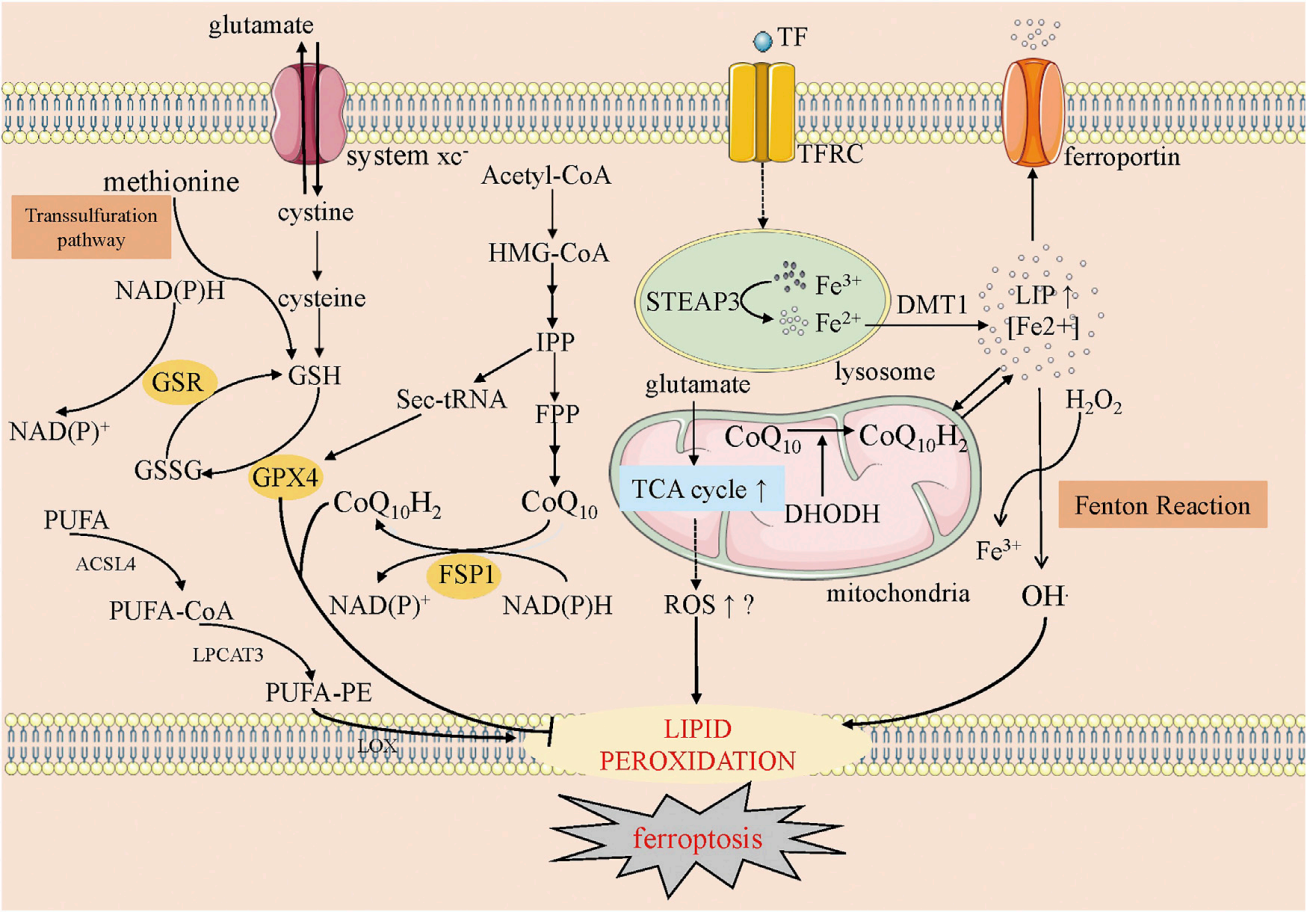
The overview of ferroptosis. This figure shows five systems regulating ferroptosis. The first one is the anti-oxidative GSH-GPX4 pathway, in which system xc^−^, transsulfuration way, GSH and GPX4 involve. The second one is the mevalonate pathway, where CoQ_10_ is produced. The third one is iron metabolism, where the Fenton reaction happens and the starter of lipid peroxidation, OH^.^, is produced. The fourth one is mitochondria, on whose membrane oxidative CoQ_10_ is catalyzed into reductive CoQ_10_ (CoQ_10_H_2_) and DHODH could inhibit this process. Meanwhile, the increased ROS along with the increased TCA cycle may help ferroptosis. The fifth one is lipid metabolism, in which PUFA is catalyzed into peroxidative lipid finally. GSH, glutathione; GSSG, glutathione disulfide; GSR, glutathione-disulfide reductase; FSP1, ferroptosis suppressor protein-1; GPX4, glutathione peroxidase 4; HMG-CoA, 3-hydroxy-3-methyl glutaryl coenzyme A; IPP, isopentenyl pyrophosphate; FPP, farnesyl pyrophosphate; TF, transferrin; TFRC, transferrin receptor; STEAP3, six-transmembrane epithelial antigen of prostate 3; DMT1, divalent metal transporter 1; LIP, labile iron pool; TCA cycle, Tricarboxylic Acid cycle; DHODH, dihydroorotate dehydrogenase; ROS, reactive oxygen species; PUFA, polyunsaturated fatty acids; PE, phosphatidylethanolamine; ACSL4, acyl-CoA synthetase long-chain family member 4; LPCAT3, lysophospholipid acyltransferase 3; LOX, lipoxygenase.

Lipid peroxidation decreases membrane fluidity, increases membrane permeability, and breaks membrane integrity. Alternatively, the decomposition products of lipid hydroperoxides, such as 4-hydroxy-2-nonenals (4-HNEs) or malondialdehydes (MDAs), may inactivate some essential proteins in many cellular processes and subsequently promote ferroptosis (Zhong and Yin, 2015) (Figure 2).

### 4.2 Regulation and Induction of Ferroptosis

Essentially, ferroptosis results from metabolic dysfunctions, including those in iron homeostasis, oxidant dysregulation, and lipid metabolism. Changes in the genes or pathways that regulate these metabolisms can alter cell sensitivity to ferroptosis.

Iron homeostasis is a physical state involving iron import, export, and storage, modulating the sensitivity to ferroptosis. An earlier study indicated that transferrin (TF), the iron-carrier protein transporting iron from the serum into the cells that has a receptor on the cell surface (TFR), is required for ferroptosis (Gao et al., 2015). It has been shown that the suppression of nitrogen fixation 1 (NFS1), a cysteine desulfurase for the synthesis of iron-sulfur clusters, can induce the expression of TFR and repress ferritin (FTH), thus sensitizing cells to ferroptosis by activating iron-starvation response (Alvarez et al., 2017). The lipogenesis regulator SREBP2 can directly induce the transcription of TF, decreasing intracellular iron pools, ROS, and lipid peroxidation, thereby conferring resistance to ferroptosis inducers (FINs) (Hong et al., 2021). Knockdown of heme oxygenase-1 (HMOX1), catalyzing the degradation of heme to Fe^2+^, decreases the labile iron pool (LIP), which is mostly in the form of Fe^2+^(Chang et al., 2018). Lysosomes are organelles that isolate and store most of the endogenous iron in cancer cells (Terman and Kurz, 2013). Disrupters such as siramesine can increase the level of reactive iron and cause ferroptosis (Ma et al., 2016). Since the first ROS was discovered in 1969 (McCord and Fridovich, 1969), it has long been known that ROS can impair chromatin, organelles, and membrane functions, eventually leading to cell senescence or death. It should be emphasized that ferroptosis is distinct from the general ROS attack process, as iron expands ROS generation via Fenton or Fenton-like reactions leading to membrane lipid peroxidation.

The transsulfuration pathway, in which methionine supplies cysteine from cystathionine for GSH synthesis, can provide intracellular cysteine when system xc-is inhibited (Hayano et al., 2016) Therefore, the GSH-GPX4 system is regarded as a classical antioxidant system in modulating ferroptosis. The co-enzyme Q10 (CoQ10)- nicotinamide adenine dinucleotide phosphate (NADPH) system is another antioxidant system linking lipid metabolism with oxidative phosphorylation in the modulation of ferroptosis. The production of CoQ10 through the mevalonate (MVA) pathway has farnesyl diphosphate (FPP) as the critical precursor of CoQ10, which is produced from acetyl-CoA through several steps (Mullen et al., 2016). Another product of the MVA pathway, Sec-tRNA, is required for selenizing GPX4 (Yang and Stockwell, 2016). The apoptosis-inducing factor mitochondria-associated 2 flavoprotein, renamed ferroptosis suppressor protein 1 (FSP1), was found to catalyze CoQ10 regeneration using NADPH; thus, this protein was able to suppress ferroptosis (Doll et al., 2019).

Remarkably, mitochondria are an important source of ROS in mammalian cells, and depleting mitochondria could rescue ferroptosis induced by cystine deprivation or erastin, but not by GPX4 inhibition (Gao et al., 2019). This is contrary to the conclusion that mitochondria depletion sensitizes cells to ferroptosis (Dixon et al., 2012). Mechanistically, this is due to the mitochondrial tricarboxylic acid (TCA) cycle and electron transport chain, which serve as the major sources of cellular lipid peroxide production during cysteine-deprivation-induced ferroptosis (Gao et al., 2019). Surprisingly, a recent study found a new pathway mediating ferroptosis via CoQ10-NADPH in mitochondria (Mao et al., 2021): dihydroorotate dehydrogenase (DHODH) was able to reduce ubiquinone (i.e., CoQ10) to ubiquinol, allowing the accumulation of this anti-oxidant inside mitochondria, and thus inhibiting ferroptosis. Notably, this pathway is parallel to mitochondrial GPX4 and independent of cytosolic GPX4 or FSP1; therefore, it is regarded as a new pathway.

Another way to prevent ferroptosis is to disrupt the synthesis of phospholipids (PLs), as it causes the loss of targets for ROS attack. Acetyl-CoA synthetase long-chain members (ACSLs) and lysophospholipid acyltransferases (LPCATs) are required for the *de novo* synthesis of fatty acids into PUFA-containing PLs. ACSLs facilitate the synthesis of PUFA-coenzyme A (PUFA-CoA), and LPCATs help transform PUFA-CoA into PUFA-phosphatidylethanolamine (PUFA-PE). It has been shown that the knockdown or loss of ACSL4 or LPCAT3 causes resistance to ferroptosis (Dixon et al., 2015). Phosphatidylethanolamine-binding protein 1 (PEBP1), a scaffold protein inhibitor of kinase cascades, binds to and directs LOX15 toward PUFAs on the cell membrane to promote ferroptosis (Wenzel et al., 2017). Ferroptosis-inducing compound 56 (FIN56), a specific ferroptosis inducer that promotes the degradation of GPX4, can activate squalene synthase. This enzyme suppresses non-steroidogenic metabolites (such as coQ10) in the MVA pathway, enhancing sensitivity to FIN56 (Shimada et al., 2016). Recently, a new pathway involving the peroxisome, ER, and a series of enzymes was found to produce lipid hydroperoxides (Zou et al., 2020). In peroxisomes, 1-O-alkyl-glycerol-3-phosphate (AGP) is synthesized from acetyl-CoA with the catalytic actions of fatty acyl-CoA reductase 1 (FAR1) and alkylglycerone phosphate synthase (AGPS); in the ER, the AGP is then converted into the significant intermediate product PUFA-plasmalogen via the enzymatic actions of 1-acylglycerol- 3-phosphate O-acyltransferase 3 (AGPAT3) and plasma phenylethanolamine desaturase 1 (PEDS1) (Zou et al., 2020; Tang and Kroemer, 2020).

### 4.3 Ferroptosis and Cancers

Several studies have indicated that some well-known cancer suppressors are involved in modulating ferroptosis. For instance, p53, a cancer suppressor mediating cell-cycle arrest, senescence, and apoptosis, also plays a role in ferroptosis regulation. The noncanonical role of p53 in regulating metabolism was firstly identified by Tongyuan Li and co-workers, i.e., p53^−/−^ mouse embryonic fibroblasts (MEFs) could produce more ROS than p53^+/+^ MEFs(Li et al., 2012). In human osteosarcoma U2OS cells, endogenous p53 repressed the expression of SLC7A11 by occupying the promoter of the *SLC7A11* gene, and cystine uptake was increased in p53^−/−^ MEFs compared to p53^+/+^ MEFs (Jiang et al., 2015). The activation of spermidine/spermine N1-acetyltransferase 1 (SAT1) expression, a transcriptional target of p53 involved in polyamine catabolism, induces lipid peroxidation and sensitizes cells to ferroptosis (Ou et al., 2016). The above two studies indicated that wild-type p53 could induce ferroptosis in cells or sensitize them to ferroptosis. However, some studies found opposite results; for example, loss of p53 could prevent dipeptidylpeptidase-4 (DPP4) accumulation in the nucleus and thus facilitate plasma membrane-associated DPP4-dependent lipid peroxidation and ferroptosis, indicating p53 restricts ferroptosis (Xie et al., 2017). Stabilization of p53 also delayed the onset of ferroptosis in response to cystine deprivation, which required the involvement of the p53 transcriptional target *CDKN1A* (encoding P21) (Tarangelo et al., 2018). Mice expressing s47, a naturally occurring p53 polymorphism in African-descent populations, showed resistance to ferroptosis induction (Jennis et al., 2016). Collectively, these results point out the need for further research on the role of p53 role in ferroptosis. In fact, P53 regulating cytotoxic T cells function is also well studied. Compared with h3T cells, h3T-*p*53 knockout T cells exhibited enhanced glycolytic ability that correlated with increased proliferation, cytolytic capacity, IFN-γ secretion, expression of stemness gene signature, and decreased TGF-β signaling, and subsequently such T cells could control murine melanoma more effectively (Banerjee et al., 2016). However, in a TME with certain tumor-infiltrating leukocytes (such as B16 melanoma), activating rather than inhibiting p53 locally could enhance CD8 CTLs and antitumor immunity (Guo et al., 2017).

Another tumor suppressor, breast cancer type 1 (BRCA1)-associated protein 1 (BAP1), encoding a nuclear deubiquitinating (DUB) enzyme that interacts with several transcriptional factors and chromatin-modifying enzymes (e.g., FOXK1/2, ASXL1/2 and OGT) (Zhang et al., 2018), has also been explored for its role in ferroptosis. In functional studies, BAP1 represses SLC7A11 expression by decreasing the monoubiquitination of histone H2A (H2Aub) on the *SLC7A11* promoter, leading to increased lipid peroxidation and ferroptosis (Zhang et al., 2018). *In vivo*, BAP1 inhibited tumor growth partly by repressing *SLC7A11* and inducing ferroptosis (Zhang et al., 2018).

In lung adenocarcinoma (LUAD), serine/threonine kinase 11 (STK11) and Kelch-like ECH-associated protein 1 (KEAP1) co-mutant status strongly predict poor survival, which is associated with significantly elevated ferroptosis-protective gene expression and less vulnerability to ferroptosis (Wohlhieter et al., 2020). Notably, NFS1 is required for protecting lung cancer cells from ferroptosis, and this requirement especially persisted in *STK11*-null cells (Alvarez et al., 2017).

As mentioned above, inducing rather than inhibiting ferroptosis has therapeutic potential for treating cancers. It is therefore essential to mention some of the main FINs, all of which used in laboratory research rather than in clinical applications. Currently, there are four classes of FINs. Class Ι FINs, widely studied in the laboratory, are based on GSH depletion and target systems xc, glutamate-cysteine ligase, glutathione S-transferase, and cyst(e)in depletion. Glutamate-cysteine ligase and glutathione S-transferase function during the formation of GSH and its conjugation with substrates, respectively. Erastin, sorafenib (an anti-cancer drug), sulfasalazine (an anti-inflammatory drug), and artemisinin and its derivatives (antimalarial drugs) belong to class Ι FINs. Class II and Class III FINs are based on GPX4 inactivation or depletion, and targets include GPX4, squalene synthase, and HMG-CoA reductase. FINs within these classes include Ras-selective lethal compound (RSL3) and FIN56, which are GPX4 inhibitors and frequently used to establish ferroptosis in research. Class IV FINs influence iron metabolism to induce ferroptosis via iron loading, iron oxidation (Gaschler et al., 2018), and increased labile iron pool. For instance, a derivative of salinomycin, ironomycin (AM5), can accumulate and sequester iron in lysosomes, where the subsequent degradation of ferritin triggers ferroptosis (Table 2).

**TABLE 2:** Ferroptosis inducers.

Mechanism	Target	Drugs/Compounds
GSH depleton (class I FINs)	system xc^-^	Erastin Dixon et al. (2012), sulfasalazine^*^, Dixon et al. (2012), glutamate^*^, Dixon et al. (2012), piperazine rastin Yang et al. (2014), imidazole ketone erastin Larraufie et al. (2015), sorafenib^*^, Louandre et al. (2013)
	glutamate-cysteine	buthionine sulfoximine Yang et al. (2014)
	Ligase	
	glutathione S-transferase	Artesunate^*^, Eling et al. (2015)
	[Cys] depletion	cyst(e)inase Cramer et al. (2017)
GPX4 inactivation (class II FINs)	GPX4	1S,3R-RSL3 Dixon et al. (2012), DPI7 Yang et al. (2014), DPI10 Yang et al. (2014)
GPX4 depletion (class III FINs)	squalene synthase	FIN56 Gaschler et al. (2018)
	HMG-CoA reductase	Fluvastatin^*^, Shimada et al. (2016), lovastatin^*^, Shimada et al. (2016)
Iron metabolism (class IV FINs)	iron loading	FeCl_2_ Li et al. (2017), hemin Imoto et al. (2018), salinomycin Mai et al. (2017)
	iron oxidation	FINO_2_ Gaschler et al. (2018)
	increasing LIP	withaferin A Hassannia et al. (2018)

[Cys], cysteine or cystine; *, FDA-approved clinic drugs.

## 5 Molecular Relationships Between Ferroptosis and ICI Therapy

### 5.1 Ferroptosis is Immunogenic

Unlike apoptosis (discussed in Section 3), ferroptosis is a form of inflammatory cell death releasing certain DAMPs; thus, it is usually classified as a type of ICD. The release of ICD markers such as DNA, ATP, and HMGB1 is likely due to the broken plasma membrane during ferroptosis, while during apoptosis the integrity of the plasma membrane is high. A recent study based on a prophylactic tumor vaccination model strongly supported that ferroptosis is a type of ICD: early, but not late, ferroptotic cancer cells (murine fibrosarcoma MCA205 or glioma GL261 cells) promoted the phenotypic maturation of bone marrow-derived DCs, and the most typical DAMPs, ATP and HMGB1, were detected along the timeline of ferroptosis (Efimova et al., 2020). An earlier study reported that extracellular HMGB1 could be released to trigger inflammation and immunity during ferroptosis induced by RSL3 and erastin (Wen et al., 2019). Notably, HMGB1 mediates inflammation during ferroptosis through the receptor binding to advanced glycation end-products (RAGE) in macrophages, whose genetic blockade can restrict ferroptosis-mediated inflammation (Wen et al., 2019). HMGB1 is also a regulator of ferroptosis, and the knockdown of HMGB1 gene can decrease erastin-induced lipid ROS generation (Ye et al., 2019). DNA is another DAMP released by ferroptotic cells, although it was identified as a tumorigenesis promoter. In a Kras-mutant mouse model, both high-iron diet and GPX4 depletion promoted Kras-driven pancreatic tumorigenesis, which was mediated by 8-hydroxy-2-deoxyguanosine (8-OHG), a major product of oxidative DNA damage, released by the ferroptotic cells, which interacted with cyclic guanosine monophosphate–adenosine monophosphate synthase (CGAS) and initiated the transmembrane protein 173-dependent DNA sensor pathway (Dai et al., 2020).

Collectively, the above results indicate that ferroptosis is an ICD. However, further research based on prophylactic tumor vaccination models (the gold standard of ICD detection) is urgently needed. ICD detection by a prophylactic tumor vaccination model has an obvious limitation: human cancer cells can trigger an adaptive immune response due to cross-species-specific immunological incompatibility and therefore cannot be directly investigated in mice models (Galluzzi et al., 2017). Nevertheless, the more rigorous the evidence supporting the immunogenicity of ferroptosis, the more tenable the anti-tumor role of ferroptosis.

### 5.2 IFN-γ at the Crossroad of ICI Therapy and Ferroptosis

The efficacy of ICI therapy is associated with preexisting intra-tumoral T-cell immunity (Tumeh et al., 2014) and activation of specific inflammatory pathways (Ayers et al., 2017). The immunogenic ferroptosis with potential ICI therapy is the latter. When ICI therapy is combined with ferroptosis induction, and given that ferroptosis is immunogenic, it is beneficial for the maturation of DCs. Moreover, blocking CTLA-4 enhances the antigen-presenting process, or the subsequent immune system attack is enhanced by blocking PD-1/PD-L1, finally establishing a stronger adaptive immune system to kill cancer cells.

The canonical IFN-γ signaling pathway (IFN-γ/JAK/STAT1 signaling pathway) was originally discovered in the 1990s. The receptor of IFN-γ (IFN-γR) is composed of two subunits, IFN-γR1 and IFN-γR2, both with intracellular carboxy termini carrying the nonreceptor tyrosine kinases Janus-activated kinase (JAK)1 and JAK2, respectively. The biologically active form of IFN-γ, which interacts with IFN-γR1 and IFN-γR2, is responsible for the intracellular transmission of the signal (Platanias, 2005) by JAK phosphorylating IFN-γR; the signal transducer and activator of transcription 1 (STAT1) and STAT2 proteins are then recruited to bind IFN-γR1 and IFN-γR2, respectively (Darnell et al., 1994). The binding of STAT1 to IFN-γR1 leads to JAK1 phosphorylation of STAT1, subsequently promoting the formation of homodimers of STAT1 that are translocated into the nucleus (Darnell et al., 1994). The promoters of downstream target genes have gamma-activated sequence (GAS) sites, where the homodimers of STAT1 bind. The major primary response of IFN-γ/JAK/STAT1 signaling is the expression of interferon response factor 1 (IRF1), which then induces several secondary response genes (Lee et al., 2013).

IFN-γ is a critical cytokine produced by activated T cells, natural killer (NK) cells, and NK T (NKT) cells in the tumor microenvironment (TME). In most circumstances, IFN-γ is regarded as an antitumor factor. IFN-γ is known to aid in the priming and presentation of antigens by upregulating MHC Ι molecules, and it is also involved in Th1-assocaited responses, which include the differentiation, activation, and homeostasis of T cells; it also mediates inflammation by activating macrophages and inducing the production of chemokines that recruit specific effector cells (Zaidi and Merlino, 2011). However, in some circumstances, IFN-γ promotes feedback inhibitory mechanisms. As a pro-inflammatory cytokine, IFN-γ can upregulate the expression of PD-L1 transcriptionally (Cha et al., 2019). In addition, tumor cells stimulated with IFN-γ can release exosomes expressing PD-L1 on their surfaces, so that IFN-γ suppresses the function of CTLs and facilitates tumor growth (Cha et al., 2019). Further evidence indicates that increased CD8 T cell infiltration and IFN-γ signaling can upregulate indoleamine 2,3-dioxygenase (IDO1) expression, promoting tryptophan (Trp) catabolism, which is known to induce numerous tolerogenic immune phenotypes (Triplett et al., 2018). This is at odds with the concept of noninflamed TMEs, defined by preexisting cytolytic CD8 T cells and low expression of PD-L1, which can impair the efficacy of ICI therapy (Kline et al., 2020). We hypothesize that the level and site of PD-L1 expression determine either anti-tumor immunity or pro-tumor immunity.

Generally, the level of CD8 T cell infiltration of tumors at baseline or on treatment is predictive of ICIs response (Grasso et al., 2020). Therefore, IFN-γ produced by activated T cells has received corresponding attention. IFN-γ signaling plays a critical role in enhancing the efficacy of ICI therapy. In a recent study on the importance of triggering IFN-γ signaling for sensitizing cells to ICIs, avadomide, a cereblon E3 ligase modulator, stimulated a feedforward cascade of revigorated T cell responses by inducing type Ι and II IFN-γ signaling in chronic lymphocytic leukemia (CLL) patients, thus leading to CD8 T cell-inflamed tumor microenvironments that responded to anti-PD-1/PD-L1 therapy (Ioannou et al., 2021). Furthermore, CD8 + T cells were activated directly through the innate IFN-γ signaling pathway. In a previous study, this effect was shown to be indirect, via DCs. Blocking PD-1 expressed on the CD8 T cell surface amplified CD8 T cell production of IFN-γ, which bound with the IFN-γR of DCs. DCs activated by IFN-γ could in turn stimulate CD8 T cells by producing IL-12, which interacted with CD8 T cells via the IL-12 receptor (Garris et al., 2018). IFN-γ signaling is also important in cancer cells. For instance, melanoma mouse models defective in IFN-γ signaling showed resistance to ICI therapy, as the tumor MHC Ι expression (which determined the efficiency of antigen presentation) depended on either type Ι or type II IFN-γ signaling (Kalbasi et al., 2020).

In conclusion, IFN-γ signaling plays an anti-tumor role during ICI therapy, which is involved in the processes of DCs maturity and CD8 T cell activation, both of which critical steps for anti-tumor immune responses. Based on this conclusion, researchers have explored whether ferroptosis is correlated with IFN-γ signaling, as such a correlation would uncover the relationship between ICI therapy and ferroptosis. In 2019, Wang and co-workers (Wang et al., 2019) creatively identified that IFN-γ sensitized tumor cells (human fibrosarcoma cell line HT-1080 and mouse melanoma cell line B16-F0) to ferroptosis by inhibiting system xc^−^. When RSL3 or erastin was combined with IFN-γ, lipid ROS in tumor cells was relatively higher than in the single-treatment groups, while the synergistic effect was low in IFN-γR1-deficient B16 cells; the tumor-burdened experiment *in vivo* led to the same conclusion. Subsequent western blotting analysis showed that IFN-γ markedly reduced tumor cells’ SLC3A2 and SLC7A11 protein expression with increased IRF1 expression, which indicated the possible involvement of the JAK/STAT1 signaling pathway. The above results provide direct evidence that IFN-γ plays a crucial role in augmenting ferroptosis by reducing SLC3A2 and SLC7A11 protein expression. In addition, PD-L1 blockade along with cyst(e)inases synergistically induced tumoral ferroptosis and increased the proportion of T cells in CD45^+^ cells and of IFN-γ^+^ T cells in CD8 T cells, which directly supported the synergistic effect of ICIs and FINs. The involvement of the IFN-γ/Jak1/STAT1 signaling pathway in ferroptosis therefore provides additional opportunities for cancer therapy, as some reagents targeting this pathway could be applied. Another study exploring the correlation between radiotherapy and ferroptosis also revealed that tumor antigen-specific CD8 T cells or IFN-γ could induce ferroptosis in melanoma cells via suppression of SLC7A11, and this effect was enhanced when radiotherapy was combined with IFN-γ (Lang et al., 2019). Moreover, a recent study indicated that a dual PI3K/HDAC inhibitor, BEBT-908, could induce immunogenic ferroptosis and promote a pro-inflammatory TME, by which immune checkpoint blockade therapy was potentiated. Mechanistically, BEBT-908-induced ferroptosis led to the activation of the endogenous IFN-γ-STAT1 signaling pathway in cancer cells (Fan et al., 2021). This study provided new evidence for the importance of IFN-γ (signaling) in the relationship between ferroptosis and ICI therapy.

TNF-α is another cytokine produced by effector CD8 T cells to augment CD8 T cell-mediated cytotoxicity beyond IFN-γ (Walczak, 2011). However, TNF-α has been proven to have no effect on increasing lipid ROS levels (Wang et al., 2019). Transforming growth factor β1 (TGFβ1), a dichotomous cytokine, acts as a tumor suppressor or tumor promoter in different circumstances. In the course of ferroptosis in hepatocellular carcinoma cells, TGFβ1 restricts the expression of SLC7A11; therefore, it drives the vulnerability of hepatocellular carcinoma cells to ferroptosis, which can be prevented by Smad3, but not by Smad2 or Smad4, revealing the involvement of the TGFβ1-Smad3 signaling pathway in ferroptosis (Kim et al., 2020). The roles of other cytokines in inducing or inhibiting ferroptosis, especially the relationship between ferroptosis and ICIs, need further research.

### 5.3 TYRO3 is Involved in ICI Therapy Resistance by Inhibiting Ferroptosis

TYRO3, a TAM (TYRO3, AXL, and MERTK) receptor tyrosine kinase (RTK), along with the cognate agonists protein S1 (Pros1) and growth-arrest-specific 6 (Gas6), plays multiple roles in certain physiological or pathological processes. For example, TAM signaling may contribute to immune homeostasis, such as the negative regulation of innate immune response, phagocytosis of apoptotic cells, and recovery of vascular integrity, while the loss of TAM signaling is associated with chronic inflammatory and autoimmune diseases (Rothlin et al., 2015).

The elimination of cancer cells by T cells is critically dependent on the optimal activity of innate immune cells (Akalu et al., 2017). The TAM system has been used to improve anti-cancer therapies, as it plays a critical role in connecting innate and adaptive immunity. It is an important negative regulator of the immune system, especially at the interface of innate and adaptive immunity (Rothlin et al., 2015). Specifically, the activation of DCs is associated with the upregulation of the TAM system, which in turn inhibits lipopolysaccharide (LPS)-induced cytokine production, such as TNF-α production (Meer et al., 2021). Inhibiting cytokine production involves triggering the expression of the suppressor of cytokine signaling proteins SOCS1 and SOCS3, which degrade adaptor molecules involved in the TLR and type I IFN signaling cascades, such as molecules those in the JAK-STAT pathway (Akalu et al., 2017). In this sense, the TAM system acts as an antigen-specific control or checkpoint in the innate immune system. Enhancing phagocytosis of apoptotic cells (also known as efferocytosis) is another anti-inflammatory mechanism mediated by the TAM system (Meer et al., 2021). For example, in a mouse model, MERTK-deficient splenic and peritoneal MΦs showed inadequate ability to phagocytize apoptotic cells (Akalu et al., 2017).

In addition to the innate immune system, TAM is highly associated with cancer cell dedifferentiation, which can lead to ferroptosis vulnerability. During acquired resistance to BRAF inhibition, the high expression of AXL, a member of TAM, is a marker of melanoma dedifferentiation, indicating these are vulnerable to ferroptosis inducers (Tsoi et al., 2018). Dedifferentiated cancer cells are more vulnerable to ferroptosis likely because they are more dependent on iron to sustain their dedifferentiated state; for example, the levels of the mitochondrial iron-sulfur cluster-containing protein aconitase 2 (Aco2) increase during the dedifferentiated state along with other enzymes involved in the Krebs cycle (Rodriguez et al., 2021). Interestingly, melanomas also dedifferentiate in response to pro-inflammatory cytokines, including IFN-γ and TNF-α, and the role of IFN-γ in sensitizing cancer cells to ferroptosis was identified as mentioned above (Tsoi et al., 2018).

In a recent study, high TYRO3 expression in tumors induced PD-L1/PD-1 blockade therapy resistance in a mouse model and in patients who received PD-1/PD-L1 blockade therapy, which was achieved by limiting tumoral ferroptosis (Jiang et al., 2021). The authors built a resistant 4T1 mammary carcinoma model that was nonresponsive to anti–mPD-1, and *Tyro3* was screened for its high expression. However, according to the bioinformatics analysis, among the three TAM RTKs, only TYRO3 is involved in anti-PD-1 resistance, while higher AXL or MERTK expression did not correlate with shorter survival times for melanoma patients who were treated with anti–PD-1. Furthermore, in a syngeneic BALB/c mouse model, anti-PD-1 treatment significantly reduced tumor growth and extended survival in mice bearing 4T1-P (anti–PD-1–responsive) tumors, but not in those bearing *Tyro3*-OE (*Tyro3*-overexpressing) tumors.

Mechanistically, the authors hypothesized that tumor cells could inhibit ferroptosis through the TYRO3-activated PI3K-AKT-NRF2 pathway to resist the antitumor activity of T cells. Genes blocking ferroptosis were upregulated (e.g., *Slc40a1, Slc7a11, Slc3a2,* and *Gpx4*) in *Tyro3*-OE 4T1 tumor cells, whereas genes that induced or enhanced ferroptosis were downregulated (e.g., *Slc5a1* and *Tfrc*), compared with levels in 4T1-P cells. These differences in gene expression could be inhibited by low expression of TYRO3 or AKT inhibitors. In addition, the HMGB1 signaling pathway (being HMGB1 a type of DAMP released by ferroptotic cells, as described above) is negatively associated with TYRO3 mRNA expression. The addition of anti–PD-1 significantly increased lipid ROS in 4T1-P, but not in *Tyro3*-OE tumor cells, supporting the assumption that TYRO3 suppresses anti–PD-1–induced tumor cell ferroptosis. According to Wang et al., 2019, T cell–secreted IFN-γ induces tumor cell ferroptosis. However, this study also presented a contradictory result: as there was no significant change in CD8 + T cell frequency or activity (using IFN-γ and granzyme B as activity indicators) between anti–PD-1–treated *Tyro3*-OE 4T1 tumors and 4T1-P tumors, it was suggested that the decrease in ferroptosis could be regulated by the intrinsic mechanisms of tumor cells.

**FIGURE 3:**
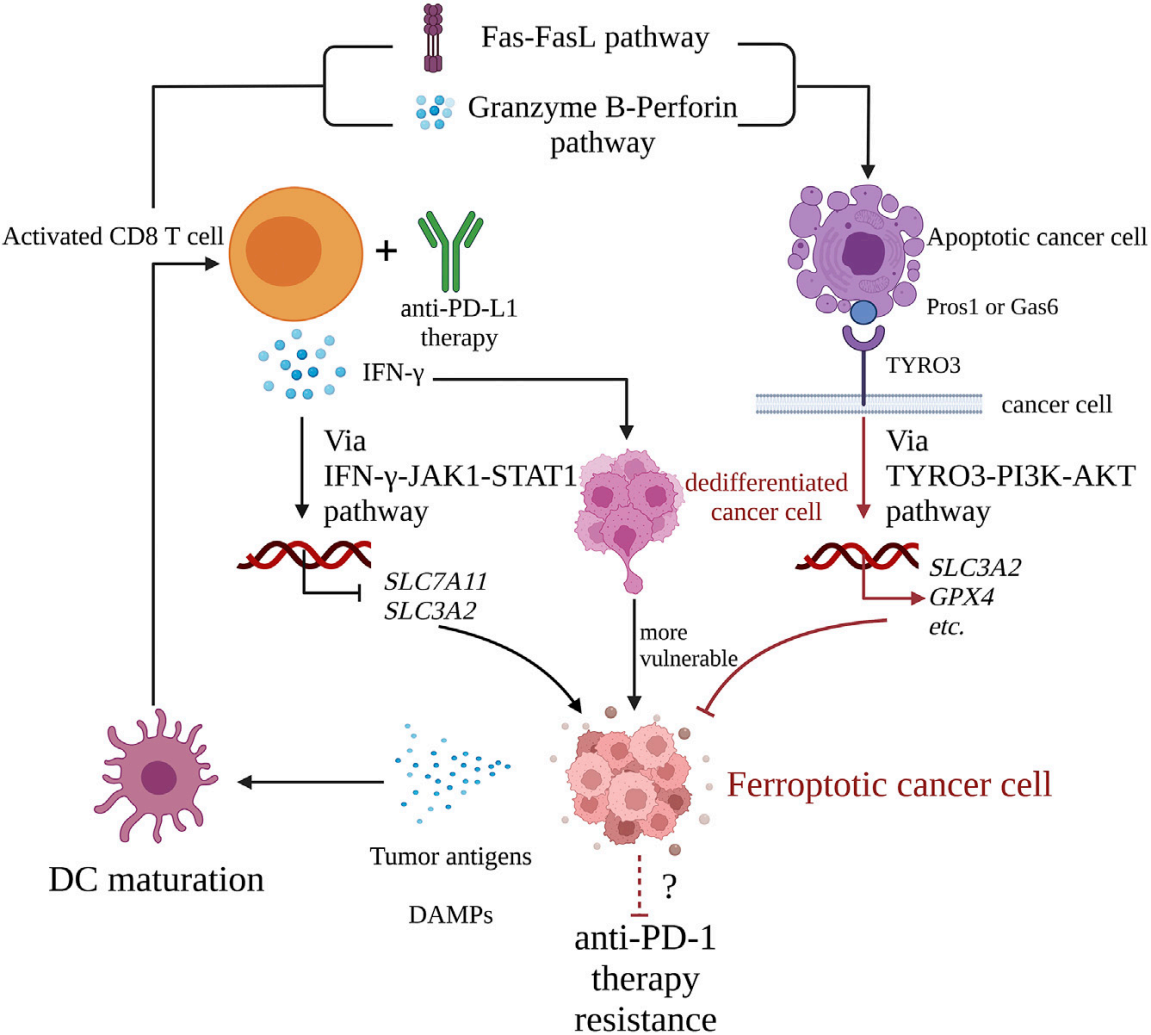
The interaction cycle of ICI therapy and ferroptosis. Activated CD8 T cells with anti-PD-L1 therapy release IFN-γ, which downregulates the expression of *SLC3A2* and *SLC7A11* via the IFN-γ-JAK1-STAT1 pathway and promotes cancer cell ferroptosis. Meanwhile, activated CD8 T cell mediates apoptosis of target cancer cell via Fas-FasL pathway or granzyme B-perforin pathway. The apoptotic cell expresses receptor tyrosine kinase TYRO3 on its membrane. Once TYRO3 binds to Pros1 or Gas6 expressed on apoptotic blebs, TYRO3-PI3K-AKT pathway is activated, which leads to upregulation of pro-ferroptosis genes such as *SLC3A2* and *GPX4*, inhibition of cancer cell ferroptosis, and cancer cells resistance to anti-PD-1 therapy. Ferroptotic cancer cells are with immunogenicity, and they release DAMPs such ATP, DNA, HMGB and so on, which promote DC maturation, a critical link of cancer-immunity cycle, and finally explaining that ferroptosis helps ICI therapy. IFN-γ, interferon-γ; JAK1, Janus-activated kinase 1; STAT1, signal transducer and activator of transcription 1; Pros1, Protein S1; Gas6, growth-arrest-specific 6; PI3K, phosphatidylinositol-4,5-bisphosphate3-kinase; AKT, protein kinase B; HMGB, high mobility group protein B; DC, dendritic cell; PD-L1, programmed cell death 1; PD-L1, programmed cell death ligand 1.

Overall, the following mechanisms might be at play: anti-PD-L1 mAb can strengthen activated CD8 T cells killing cancer cells in ferroptosis via the IFN-γ-JAK1-STAT1 signaling pathway; DAMPs released by ferroptotic cells help in DCs maturation, which induces CD8 T cell activation; activated CD8 T cells then mediate cancer cells apoptosis, inhibiting ferroptosis through binding of TYRO3 and Pros1 or Gas6. However, in the present review we focused only on cancer cell death and not on CD8 T cell death Figure 3.

## 6 Concluding Remarks

Over the past decade, considerable progress has been made in ferroptosis research, involving different research fields but especially cancer and immunology research. Ferroptosis is a new type of regulated cell death. Its essence is the uncontrolled lipid ROS mediated by ferric ion-associated Fenton-reactions attacking biological membranes and leading to lipid peroxidation. Several studies have identified correlations between ferroptosis and cancers. As mentioned above, a classical tumor suppressor, p53, repressed the expression of SLC7A11 by occupying the promoter of the *SLC7A11* gene. In addition, *STK11* and *KEAP1* co-mutant status could promote ferroptosis-protective gene expression. These correlations between ferroptosis and cancers highlight the role ferroptosis may play in cancer therapy. In fact, the achievements of crossover research on ferroptosis and radiotherapy are far more than those of ICI therapy. The most common link between ferroptosis and radiotherapy is the activation of ROS, which has been well summarized in a recent review (Lei et al., 2021). As for the link between ferroptosis and ICI therapy, this are harder to hypothesize than that between ferroptosis and radiotherapy; in other words, the activation of ROS as the link may not be applicable.

However, bidirectional relationships have been identified. Ferroptosis might enhance the efficacy of ICI therapy via its immunogenicity in the anti-tumor immune response. Unlike apoptosis, ferroptosis is a type of ICD. Efimova et al. identified that ferroptotic cancer cells promoted the phenotypic maturation of bone marrow-derived DCs. As the maturation of DCs is a critical point of the cancer-immunity cycle for ICI therapy, the nature of immunogenic ferroptosis indicates its potential to enhance the efficacy of ICI therapy. On the other hand, ICI therapy can promote ferroptosis in tumor cells. A study by Wang et al first uncovered the correlation between ferroptosis and ICI therapy and found that IFN-γ sensitized tumor cells to ferroptosis by inhibiting system xc^−^, and PD-L1 blockade plus cyst(e)inase synergistically induced tumoral ferroptosis. This study strongly indicated that PD-L1 blockade could kill tumor cells through ferroptosis via IFN-γ signaling rather than other kinds of RCD. The opposite logic is that the efficacy of ICI declines when ferroptosis is inhibited. A molecule of the TAM system, TYRO3, functions in such a logic. A study by Jiang *et al* showed that high expression of TYRO3 could induce tumor cell resistance to anti-PD-1/PD-L1 therapy by upregulating genes that block ferroptosis and downregulating genes inducing ferroptosis, thus inhibiting ferroptosis. We hypothesize that ferroptosis in the TME may be associated with the efficacy of ICI therapy, with the research results reported above supporting this hypothesis mechanistically.

Beyond the existing concepts and results of previous studies presented in this review, some old and new issues deserve further exploration: 1) The efficacy of ICI therapy is influenced by the expression level of checkpoints to some extent; thus, it should be explored if there are any correlations between the expression level of checkpoints and the frequency of ferroptosis; 2) A preliminary study exploring immunogenic ferroptosis has been conducted but more evidence based on prophylactic tumor vaccination models (the gold standard of ICD detection) in multiple circumstances is needed to support the immunogenicity of ferroptosis; 3) Although IFN-γ and TYRO3, and the molecular mechanisms they are involved in, were found to upregulate or downregulate ferroptosis, more molecules and pathways should be endowed with similar roles, and therefore investigated as possible links between ICI therapy and ferroptosis; and 4) Anti-CTLA-4 therapy is another main constituent of ICI therapy with different mechanisms from anti-PD-1/PD-L1 therapy but there is little cross-research between anti-CTLA-4 therapy and ferroptosis. Furthermore, beyond PD-1/PD-L1 and CTLA-4, research on the crosstalk between novel immune checkpoints and ferroptosis is also a totally new field worth further exploring.

This review was restricted to the research uncovering the complex connections between cancer ICI therapy and cancer cell ferroptosis. In addition, several studies revealed the adverse effect of ferroptosis on ICI therapy, although this was not discussed in this review. Finally, we only reviewed the molecular links between ICI therapy and cancer cell ferroptosis and did not explore the role of ferroptosis in TME in detail, along with the influence of immune cells ferroptosis. For example, a recent study showed that cholesterol in the TME could trigger CD8 T cell engulfing of extrinsic lipids, leading to CD8 T cell ferroptosis; as a result, CD8 T cell released less IFN-γ and showed a low kill energy (Ma et al., 2021). Associated information about ferroptosis in TME has been thoroughly reviewed (Xu et al., 2021).

In summary, the cross-research of ICI therapy and cancer cell ferroptosis is a new field with great potential for overcoming resistance to ICI therapy, broadening the indications of ICI therapy, and promoting the clinical use of ferroptosis. However, more evidence is urgently needed to blueprint this new field.
